# An Ectopic Breast Tissue Presenting with Fibroadenoma in Axilla

**DOI:** 10.1155/2013/947295

**Published:** 2013-03-27

**Authors:** Anandhi Amaranathan, Kanchana Balaguruswamy, Ramachandra V. Bhat, Manash Kumar Bora

**Affiliations:** ^1^Surgery Department, Indira Gandhi Medical College and Research Institute, 225, Vazhudavur Road, Kathirkamam, Pondicherry 605009, India; ^2^Surgery Department, Aarupadai Veedu Medical College and Hospitals, Kirumambakkam, Pondicherry 607402, India; ^3^Pathology Department, Indira Gandhi Medical College and Research Institute, 225, Vazhudavur Road, Kathirkamam, Pondicherry 605009, India; ^4^Department of Radiodiagnosis, Aarupadai Veedu Medical College and Hospitals, Kirumambakkam, Pondicherry 607402, India

## Abstract

*Introduction*. The congenital anomalies of breast, especially the polymastia (supernumerary breast) and polythelia (supernumerary nipple), always do not fail to amuse the clinicians because of their varied presentations, associated renal anomalies, and pathologies arising from them. The axillary polymastia is a variant of ectopic breast tissue (EBT). Ectopic breast tissue can undergo the same physiological and pathological processes as the normally located breast. The incidence of fibroadenoma developing in ectopic breast is reported as a rare entity, the most common being the carcinoma. *Case Presentation*. A 31-year-old Dravidian female presented with a lump of 4 cm in the right axilla for the past year which gradually increased in size, giving discomfort. Our initial differential diagnosis was fibroadenoma, lipoma, and lymphadenopathy. Further investigation and histopathological report of excision biopsy confirmed it as a fibroadenoma on ectopic breast tissue in the axilla. Patient has no associated urological or cardiac anomaly. *Conclusion*. This case has been reported for its rarity and to reemphasise the importance of screening of EBT for any pathology during routine screening of breast.

## 1. Introduction

The incidence of supernumerary or ectopic nipple is around 1–5% and even less is the incidence of ectopic breast tissue (EBT) [1]. Pathologies developing in an EBT are reported as a rare entity in the literature. Carcinoma is reported as the common pathology followed by inflammation and fibroadenoma [2, 3].

We report a case of fibroadenoma in the axillary polymastia for its rarity to emphasise the importance of considering the ectopic breast and its associated pathology in the differential diagnosis of axillary mass and also to stress the importance of evaluating the patients to rule out renal anomalies or urological malignancies as it is an important association [4].

## 2. Case Presentation

A 31-year-old Asian female came presenting with the complaints of mass in the right axilla for one year which is gradually increasing in size and associated with pain and discomfort.

### 2.1. Clinical Examination

**Figure 1:**
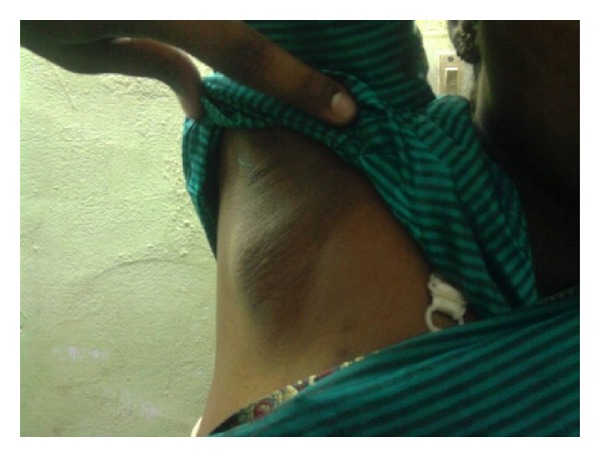
Clinical picture showing the swelling in the axilla.

On examination, 4 × 4 cm size swelling is noted in the right axilla (Figure 1). It is firm in consistency, nontender, freely mobile, and completely separate from the right breast. Skin over the swelling is normal, with no nipple or areola made out. Both the breast and left axilla are clinically normal. A provisional differential diagnosis of fibroadenoma, lipoma, and lymphadenopathy was made.

### 2.2. Investigations

**Figure 2:**
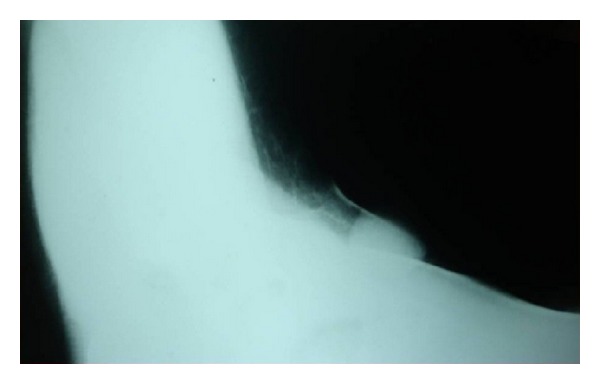
Mammographic picture showing the space occupying lesion in the axilla.

Ultrasonogram of the local parts showed 2.8 to 1.6 cm space occupying lesion (SOL) in the right axilla with well-defined and smooth margins and homogenous, hypoechoic internal echoes were noted. Color flow Doppler study showed low vascularity in the lesion, characteristic of fibroid. Both breasts are normal. Ultrasonogram of the abdomen showed no renal anomaly. Mammography of the right breast is normal. The visualized right axillary region reveals a focal bulge of homogenous soft tissue density of size approximately 2 × 2 cm, suggestive of benign SOL in axilla fibroadenoma (Figure 2). Fine needle aspiration cytology (FNAC) report showed branching monolayered sheets of benign ductal cells, bare nuclei, and occasional stromal fragments in a haemorrhagic background suggestive of fibroadenoma.

### 2.3. Treatment

**Figure 3:**
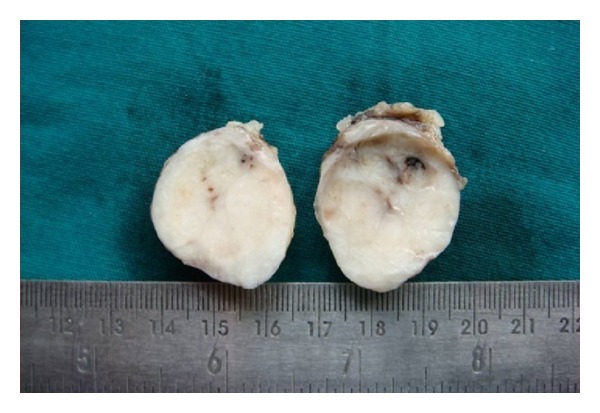
Excised gross cut section of the fibroadenoma.

**Figure 4:**
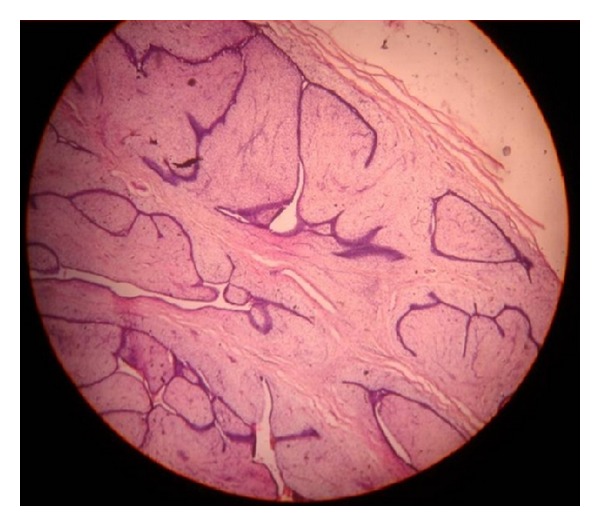
Microscopic picture of the specimen resected.

Patient underwent excision biopsy (Figure 3). Per operative finding was a subcutaneously located lesion which was excised. This anatomically superficial location of the lesion explains why this is an example of ectopic breast tissue rather than an extension of breast parenchyma into the axilla (axillary tail of Spence) which is located deep. Histopathological examination of the resected specimen (Figure 4) is also suggestive of fibroadenoma in ectopic breast tissue and the surrounding tissue shows features suggestive of breast tissue.

## 3. Discussion

Polymastia is a term that is used to describe the presence of more than two breasts in human beings. It is synonymous with supernumerary breast, accessory breast, and ectopic breast tissue (EBT).

During the 6th week of embryonic development, the mammary milk lines, which represent 2 ectodermal thickenings, develop along the sides of the embryo, extending from the axillary region to the groin. In normal development, most of the embryologic mammary ridges resolve, except for 2 segments in the pectoral region, which later become breasts. Failure of any portion of the mammary ridge to involute can lead to ectopic breast tissue with (polythelia) or without (polymastia) a nipple-areolar complex. Therefore, ectopic breast usually occurs along the “milk line” or mammary line [5].

Ectopic breast tissues are reported in locations other than the milk line, face [6], foot [7], lumbar region, vulva [8], and perineum. Supernumerary tissues present in any location other than along the milk line are supported by two beliefs. One is that it represents a migratory arrest of breast primordium during chest wall development [9]; the other belief is that it develops from the modified apocrine sweat glands [10].

In 1915, Kajava published a classification system for supernumerary breast tissue that remains in use today. Class I consists of a complete breast with nipple, areola, and glandular tissue. Class II consists of nipple and glandular tissue but no areola. Class III consists of areola and glandular tissue but no nipple. Class IV consists of glandular tissue only. Class V consists of nipple and areola but no glandular tissue (pseudomamma). Class VI consists of a nipple only (polythelia). Class VII consists of an areola only (polythelia areolaris). Class VIII consists of a patch of hair only (polythelia pilosa) [11]. Our case belongs to class IV.

Usually ectopic breast tissue occurs sporadically, but a hereditary predisposition has also been reported [12]. In most cases, accessory breasts are asymptomatic and cause nothing more than a visible distension which may resemble a tumour. Sometimes it could cause psychological disturbances in adolescence and it may give pain and discomfort especially during menstruation, pregnancy, and lactation [5]. The clinical significance of the polythelia and polymastia lies in the fact that apart from the psychological and cosmetic impact, it develops the same pathological changes as the normally located breast tissue such as inflammation, fibrosis, fibroadenoma, cystosarcoma phyllodes, and carcinoma [3, 5]. Usually carcinoma arising from the ectopic breast presents late with poorer prognosis due to delay in the diagnosis. This delay happens due to a broad differential diagnosis for an axillary lesion, including lipoma, sebaceous cyst, vascular lesions, suppurative hidradenitis, cat scratch disease, lymphadenopathy, secondaries in lymph nodes, tuberculosis, axillary tail of Spence, or even a torn muscle belly and malignancies [13].

The next important point is that these ectopic breast tissue patients, especially polythelia cases, have been associated with urinary abnormalities such as supernumerary kidneys, failure of renal formation, renal adenocarcinoma, hydronephrosis, polycystic kidney disease, duplicate renal arteries, and ureteric stenosis. This association can be partly explained by the parallel development of mammary structure and genitourinary system [4]. Even though this association needs further studies to say more about the exact frequency of association, precautionary measures should be taken in these patients to rule out any renal problem.

If EBT is associated with any suspicion of pathology, then further investigation with FNAC, ultrasonogram, mammography, and biopsy should be done as for any other breast lesion [8]. In routine screening programmes for breast cancer, a clinical examination should be made for the presence of EBT, and, if present, that should be subjected to routine screening as well, along with the normally positioned breast.

## 4. Conclusion

In conclusion, When tumors or nodules are found along the mammary line, the presence of breast tissue should be considered during the investigation [3]. It is clinically wise to evaluate and screen carefully cases of supernumerary breast for any pathology and for any associated urogenital anomalies. FNAC is very valuable in diagnosing the lesion in EBT. The treatment options for EBT depend upon the psychological factors, symptoms, and the presence of pathology. In our case, excision of the fibroadenoma has been done and the patient is on regular followup.
